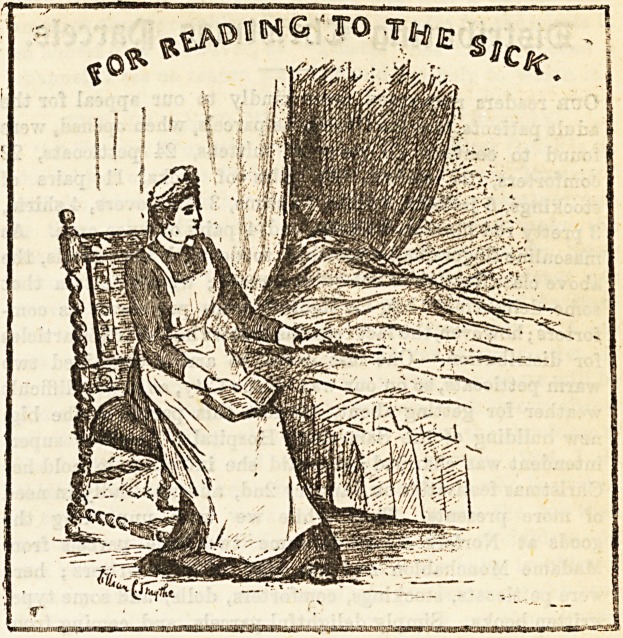# Extra Supplement—The Nursing Mirror

**Published:** 1891-01-03

**Authors:** 


Being the Extra Nursing Supplement of "The Hospital" Newspaper.

All Contributions for this Supplement should be addressed to the Editor, The Hospital, 140, Strand, London, W.C., and should have the word "Nursing" plainly written in left-hand top corner of the envelope.

### En Passant.

St. Helena Home.—This Home sends us an ancient report with certain passages marked; we know not why or wherefore. But the tale of the Home is worth telling. The Home was started in 1883 as an association not for profit, and any surplus was to be used in supplying pensions for the nurses. The Home slowly prospered; patients were received; nurses were sent out; and all went "merry as a marriage bell." But the nurses did not make use of the pensions, for they seldom stayed long in the service of the Home. Private nursing grows wearisome after a certain time. So quite lately the Home has decided to award the profits in a yearly bonus to each nurse according to length of service. We doubt the wisdom of this: the nurse receives her bonus, bids the Home adieu, and spends her money. It would be much wiser for the Home to affiliate with the Pension Fund. Congratulations are certainly due to the Committee for the success with which they have worked their scheme.

Short Items.—Huddersfield Nurses' Home has only been established five months, but already seems to have taken root, and more nurses are wanted.—The East London District Nursing Society has affiliated with the Queen Victoria Institute.—The Workhouse Infirmary Nursing Association is supplying the nursing staff for the new Hartlepool Union Infirmary.—Miss Twining has an interesting paper on training for insane cases in the *Nineteenth Century* for December; we recommend it to attendants.—One of the Winchester sisters who has worked at the County Hospital since 1866 has received an appointment at St. Thomas's, the first time, we believe, that a nurse not of Nightingale training has been taken on the staff.—Mrs. Macdonald (late Sister George) was last week married to Mr. Maurice Eden Paul.—Sister Frost desires to thank all those who have sent her Christmas greetings.—Ober-Amergau means to devote some of the wealth acquired by the Passion Play to endowing a fund to supply nurses to a hospital they intend to build in the village.

Looking Forward.—And now one word to wish all our readers a Happy New Year, and to thank those who have sent us such kindly Christmas greetings. Each year the bond grows closer between us all, and we gain new friends where the truest friends are ever to be found—in the nursing ranks. It is a delightful thing this feeling of unity, this contemplation of the gathering of nurses together in one great sisterhood, where pettiness and jealousy are not allowed to come. Happy, indeed, will the new year be if all through its days we can remember the Christmas chime of "peace and good-will." Life is too short for remembering trifling ills or indulging in little tempers: not many more years may be granted to some of us. In the words of a modern poet:—

"Adrift on Time's returnless tide,
As waves that follow waves, we glide.
God grant we leave upon the shore
Some waif of good it lacked before;
Some seed or flower or plant of worth,
Some added beauty to the earth;
Some larger hope, some thought to make
The sad world happier for its sake."

Sweet Innocence.—We do not remember ever having seen a more delicious paragraph than the following, occurring in an account of the proceedings of the Brentford Board of Guardians: "The Appointment of Night Nurse.—The Board proceeded to deal with a couple of applications for the post made vacant by the resignation of the night nurse. One of the applicants stated that she had just seen that there were 150 cases in the house, and she felt she could not conscientiously perform the duties connected with so many patients. The appointment, therefore, fell upon Mrs. McWilliams, formerly of Torrington, who said had formerly read with the object of taking medical examinations."

New York Hospital.—We have received the fifteenth annual report of the New York City Training School for Nurses, which, we are pleased to see, is printed at the Insane Asylum. The work of the Training School steadily increases, and now embraces the nursing of five distinct hospitals; the work is done by sixty-four pupil nurses and thirty-four permanent nurses. The superintendent is Miss Louise Darche, who signs the report sent. The nurses are on duty from 7.30 a.m. to 7.30 p.m., with an hour for dinner, and, when hospital duties permit, additional time for rest and study. They are also given a half-day every week, and when possible every second Sunday. A vacation of two weeks is allowed each year. Courses of lectures are given, and every year a list of nurses who have "graduated," or finished their training, is printed; last year eighteen nurses graduated.

Eighteen-Ninety.—In saying Good-bye to the Old Year it is well to glance at the work done. Last January Sister Rose Gertrude sailed from Liverpool for Molokai; the Butterworth medals for Guy's nurses were first presented; an improved nursing system was started at the South Hants Infirmary; trained night nurses were appointed at Cardiff Union. In February a new District Nursing Institution was established in Haggerston; Miss Louisa M. Gordon was appointed matron of St. Thomas's; Nurse Porter, of Edinburgh Royal Infirmary, died. In March trained nursing was inaugurated at the Sheffield Union Infirmary. In April died Mr. Junius S. Morgan; the Brassey Holiday Home was opened, also a Nursing Institution at Richmond, Surrey; the Bill for the Registration of Midwives was brought before Parliament. In May the Devonshire Square Institution celebrated its fiftieth anniversary. In June the Hospital for Sick Children, Great Ormond Street, undertook to pay its private nurses a percentage on their earnings. In July the Princess of Wales gave a reception at Marlborough House to the First Thousand nurses who had joined the Pension Fund, and presented each with a certificate. Charges were brought against the nursing system of the London Hospital by a few witnesses before the Lords' Committee, and refuted by Sir Andrew Clark, Mr. Treves, Miss Manley, and others. The Huddersfield Nurses' Home was started. Trained English nurses were sent out to Hong Kong Government Hospital. In August the Surbiton Nursing Institution was opened, and Miss Kate Marsden, trained nurse, started for Russia to study leprosy. In November died Lady Rosebery, ever a true friend to nurses. In December the Junius S. Morgan Benevolent Fund, a branch of the Pension Fund, was made up to £10,000.

### Lectures on Surgical Ward Work and Nursing.

By ALEXANDER MILES, M.B. (Edin.), C.M., F.R.C.S.E.

### LECTURE VIII.—WOUNDS AND CLEANLINESS.

PERSONAL CLEANLINESS.—As it is the nurse's duty to hand up the dressings to the doctor, it is of the utmost importance that she should be "surgically clean." By this term is meant that she should be free from every kind of septic germ, which, by gaining access to the wounds, might set up putrefaction. If then she has been working with anything septic, or likely to be a fruitful source of sepsis during the day, such as a septic abscess or ulcer, she should change her apron before assisting at an aseptic dressing. Under all conditions she must purify her hands. Mark, I do not say "wash" her hands, that is not sufficient for surgical purification. Let her carefully purify with soap and tepid water, freely using the nail brush, and then thoroughly rub the hands all over with spirits of turpentine, which is a powerful antiseptic and is not injurious to the skin. Then dry with a fresh clean towel.

If the nurse has occasion to touch the limb, especially if near the wound, or any dressing or instrument which goes into absolute contact with the wound, she must not neglect first to dip her hands. This word, "dip" is rather unfortunate in this connection. Too often the hands are only dipped. It is not uncommon to see a nurse put the points of her fingers into the lotion pretty much as she would use her finger-glass after dinner, and suppose that they are purified. Not so; the hands should be thoroughly rubbed over with the lotion by means of a swab of wool. Do not think that I am carrying antiseptics too far in insisting on these minutiæ, because if we are going to adopt antiseptic principles at all, no details are too insignificant to claim our attention. If it is necessary that the nurse's hands should be pure, it is evidently at least equally important that the surgeon's should be. It is the nurse's duty to see that the necessaries for this being done are at hand. A liberal supply of tepid water, soap, a nail brush, turpentine, and a clean towel. Never set down cold water for this purpose, even in warm weather, because, in the first place, it is not so good for cleaning the hands, and, secondly, it cools them down and renders them uncomfortable to the patient.

THE LOTION.—It will depend on the nature of the wound and the tastes of the surgeon what particular lotion is used in each case. Let us suppose the Corrosive Sublimate (1 in 2000) is what is wanted. Take two enamelled lotion basins, and put into each a quantity of 1 in 1000 corrosive, then add an equal quantity of tepid water, thus bringing it down to the proper strength. Don't add more than half fills each basin. Tepid water is added in preference to cold, being more comfortable for the patient. Into one basin put a small amount of corrosive wool to use as a swab for washing the wound. Sponges should never be used for ward dressing, because, as they are used for all sorts of cases, they become simply a vehicle for spreading sepsis. The second basin of lotion is to be used for syringing out the wound, and into it you must put nothing. If carbolic lotion (1 in 40) is used, dilute 1 in 20 with equal parts of tepid water, just as you do with corrosive; but with boracic it is better to heat the full strength lotion rather than to further dilute it with water.

SYRINGING OF WOUNDS.—It may be convenient here to say a few words on this subject. I will not discuss the advisability of doing it, but will simply give you some directions as to how to do it. There are many kinds of syringe available, but perhaps the best for all purposes is the Higginson. By it you can get a constant stream, the strength of which can readily be varied, and the flexible tubes allow it to be applied in any direction. Of the barrel syringes, perhaps the best is that made of glass. It is cheap, can be used for corrosive sublimate, is easily kept clean, and the presence of air in it is readily detected. Whichever kind of syringe you use, this subject of air is of much importance. Before introducing the nozzle of the syringe into your wound, you must make sure that all the air has been expelled, as air introduced to the wound may carry with it the elements of putrefaction. With the Higginson's syringe, the best way to make sure of the absence of air is to have the weighted end of the tube in a considerable depth of lotion, and never to let the lotion get below the level of the weight. First expel all air from the tubes by running a stream through for a little time. With the barrel and piston syringe fill it very slowly, and when the piston-rod is withdrawn to its full extent, hold the nozzle straight up in the air. This enables what air is in the barrel to rise to the surface. Now push up the piston-rod till a jet of fluid escapes, and expels the air in front of it. Don't be deceived by a small jet which often comes at the very first push in a badly working syringe; wait till a ful stream comes from the nozzle. Having filled the syringe and expelled the air, place it in the second basin with the nozzle well under the lotion, so that no more air may enter. In using the syringe, introduce the nozzle to the deepest part of the wound, so that the direction of the stream is from within outwards.

REMOVAL OF OLD DRESSING.—Having thus prepared the patient and all the requisite materials for dressing, the next stage of necessity is the removal of the old dressing. I need not insist on the importance of gentleness in this, not only to avoid causing the patient unnecessary pain, but also to prevent undue movement of parts in which the maintenance of rest is of immense importance in the treatment. It will often be your duty to steady the patient's limb during dressing. Always purify your hands before doing so, and try to hold the part in as comfortable a position as possible. Perhaps the most comfortable way for the patient in holding a leg is to seize it by the great toe, although it is rather tiresome for the nurse. The domette bandage should be rolled off by simply reversing the movement of putting it on. Never remove a bandage by seizing the end, and then describing circles round the patient's foot, coiling up the bandage into a rope. The deeper bandages may be cut off, as they are not to be used again. Cut along the front of the limb by short snips of sharp scissors. Then comes the wool. This should be taken off, as it was put on, in layers, taking care not to expose the wound till you are ready to irrigate it with lotion. You will find that wool is more easily removed dry than wet; hence, you should never put lotion on to the wool, unless it is sticking into the wound. In removing the deep dressing raise the upper edge, and by a stream of lotion from a gradually float up the rest.

DRAINAGE TUBES.—These are usually removed at each dressing and cleansed, and then reintroduced or not, according to circmmstances. When removing a drainage tube always carefully note the exact direction it takes in the wound, so that you may know how to reintroduce it. Should you experience any difficulty in doing this, a probe passed into the lumen of the tube, making it stiff will often greatly facilitate its passage. Always take some means of preventing your drainage tube slipping right into the wound, especially when dealing with cavities like the pleura or peritoneum. Even in quite superficial wounds, indeed, masses of granulations sometimes grow up, completely concealing the end of the tube, which might easily be overlooked. Perhaps the simplest method is to transfix the tube with a sterilised safety pin. It is a safe rule to remove, or at least shorten, a tube, when the wound forms an exact mould of it.

*(To be continued.)*

### Nursing Medals and Certificates.

No. III.—THE MARY ADELAIDE MEDAL.

THIS medal is for nurses belonging to the Workhouse Infirmary Nursing Association. It was first presented in 1882. The device was designed by Lord Montagu, and the die presented by H.R.H. Princess Mary Adelaide, Duchess of Teck, whose initials "M. A." form part of the design. In 1881 H.R.H. had allowed her name to be given to nurses working under the association. The medal is given to probationers trained by the association after one year of satisfactory service; to nurses who receive training from other institutions it is given after two years' satisfactory work in one situation. The medal must be returned when a nurse ceases to work under the association, and the following are the names of those who hold the medal: Nurse Ellen Murray, Nurse M. Thompson, Nurse Harnden, Nurse Isabella Tilbury, Nurse Pinnington, Nurse Christine Foster, Nurse A. Blackwell, Mrs. J. Jackson (trained by the association), Mrs. E. Jackson, Nurse Woolley, Nurse Costin, Nurse Wright, Nurse Hudson, Nurse Stevens (trained by the association and Mrs. Radford), Nurse Allen (trained by the association), Nurse Annie Roberts, Nurse Mary Ann Jones, Nurse E. Boddy, Nurse A. E. Vanse (trained by the association), Nurse Adam (trained by the association), Nurse E. Maxwell, Nurse L. Winter, Nurse M. Guernsey, Nurse E. Pendrill (trained by the association), Nurse C. Süche, Nurse R. Millage (trained by the association), Nurse M. Donovon (trained by the association), Nurse A. Britt (trained by the association), Nurse E. Gold (trained] by the association), Nurse M. Bleaney (trained by the association), Nurse M. Rogers (trained by the association), Nurse R. A. Rose (trained by the association), Nurse C. Blackwell (trained by the association), Nurse M. King, Nurse E. Flawn (trained by the association), Nurse E. Allen (trained by the association), Nurse L. Matthews (trained by the association), Nurse Pink (trained, by the association), Nurse Walsh, Nurse Bassett, Mrs. Cant, Mrs. Ross, Nurse Saunderson, Nurse Pruett, Nurse F. Cooke, Mrs. Key, Nurse K. Ferguson, Nurse L. Waite (all trained by the association), Nurse A. Smallhorn (trained by the association).

Perhaps no nurses better deserve a medal than those working for the Workhouse Infirmary Nursing Association. They are fighting the battle in infirmaries which Miss Nightingale and her compeers fought in hospitals, and though in many infirmaries there is now an adequate nursing system, in many there is no system whatever, and the trained nurse has to toil on without proper tools and in the face of ignorant opposition. It is superfluous in these pages to praise this association, we all know its good work so well; we are sure all our readers wish it a successful and happy New Year.

### SNOW.

IT seems cold comfort at first to talk about snow in bitter weather, but the thing itself is so beautiful that we can but look at and admire the lovely feathery flakes as they fall, and we may also get some comforting thoughts if we look for them in the right way. We read, "God sendeth forth His snow-like morsels, and who is able to abide this frost?" Yet, in common with all His gifts, they are both for a good purpose. Frost kills the insects which would destroy the crops, and then the snow covers over the earth with a garment of dazzling white, which warms and preserves the roots and bulbs that would otherwise perish. How lovely the country looks with trees and shrubs enveloped with snow, every branch traced out in delicate hoar frost. Hill and dale are one uniform white, and the whole scene speaks of purity and peace. Slowly and silently the world has put on her thick, warm covering, and the vegetation is preserved; then in the spring, when the sun gains power, it melts away, and every plant shoots up again and makes the land bright and gladsome.

There are many beautiful promises to men in the Bible, and none more so than when the Holy Spirit says by the Prophet Isaiah "Though your sins be as scarlet they shall be white as snow." As snow falls silently on the earth and changes the whole face of nature, so the love of God lets the precious blood of Jesus drop gently on the sinner's soul, covering it with a robe of purity, and making it clean and white in His sight. Then as the earth is warmed by snow so God's love warms our hearts, cherishing the roots and seeds of good desires which have been sown in them, and when the Sun of Righteousness shines upon us we shall bring forth fruit to eternal life.

With purity comes peace,' that peace of God which passeth all understanding. What a hush and stillness there is when snow is around us! It deadens the sounds of human wrangling and discord, the very traffic of the world seems far away. So when God's love and peace wrap the soul all else appears worthless. To live and die in His protecting arms, to leave all to Him who knows how to take care of us, is what we desire alone. When we see snow falling we will try to remember that He who sendeth it can cover us with the righteousness which will protect us from the cold blast of unbelief. Oh! long for the white garment which is the righteousness of the saints and which is given to those who have kept themselves pure by the help of the Holy Spirit.

### Distributing Christmas Parcels.

OUR readers responded most kindly to our appeal for the adult patients, and the Christmas parcels, when opened, were found to contain 25 pairs of mittens, 24 petticoats, 22 comforters, 21 shawls, 20 pairs of socks, 11 pairs of stockings, 6 jackets, 8 dolls, 7 aprons, 3 cross-overs, 4 shirts, 3 pretty red blouses, 4 books, and 4 pairs of knee-caps. As masculine fingers had somewhat to do with the parcels, the above classification is not quite correct; we are certain that some delightful "hug-me-tights" were put down as comforters; however, the fact remains that we had over 150 articles for distribution. One last parcel to arrive contained two warm petticoats, so on our way to the City, as it was difficult weather for getting about, we left this parcel at the big, new building of the Samaritan Hospital. The lady superintendent was charmed; she said she intended to hold her Christmas festivities on January 2nd, and was sadly in need of more presents. Then while we were unpacking the goods at Norfolk House in came two huge parcels from Madame Monchablon and her staff of type-writers; here were petticoats, stockings, comforters, dolls, and some type-written books. Simply delightful parcels, and coming from busy hands they were all the more appreciated.

It was with some difficulty we found a cabman brave enough to face the fog and drive us and our impedimenta about the dark, slippery streets. That cab, laden with brown paper parcels, both inside and out, must have presented a queer appearance. First of all to King's College Hospital, where the sister-matron received, with warmest thanks, three of these parcels; we pass the thanks on to those who worked the garments, and wish we could make them hear the really hearty tone of gratitude with which they were uttered. Then down to Charing Cross, where we learnt with regret that the lady superintendent was ill; the parcels were, therefore, left with the secretary, and in the morning came a formal vote of thanks from the Weekly Board. Next to Westminster, where Miss Pyne seemed very busy with Christmas preparations, and surrounded with gifts for the patients. Then on to the Middlesex, where we left some parcels of women's clothing for the Queen wards. Then to Bloomsbury Square, where we handed over to Miss Mansel 13 bed-jackets from the new competition; these, she declared, would be charming gifts for her patients, and particularly pleased was she with the jackets which opened on shoulders and down the sleeves, as she had several cases of rheumatism, where the least movement caused pain. We also got Miss Mansel to kindly send on six bed-jackets to the South London Nursing Society, as owing to the weather it was very difficult to get the parcels distributed. Three jackets we sent by post to the matron of the Lyme Regis Cottage Hospital, at her special request; but other requests from Chichester and Southampton for Christmas parcels we were unable to comply with, as we had stated that the garments were for distribution in the metropolis. The two prize jackets we have retained for the present to take the pattern.

So one cab load was gone, the next we took East to the London Hospital, where we left a few things for men in the Harrison ward, and a few things for women in the Charlotte ward. It was useless here to try and distribute over the whole hospital. We hope next year we shall be more able to meet the great need for warm garments. We could have done with 550 instead of 150. A nice big bundle of bed-jackets for the East London Nursing Society; three parcels put aside for St. Mary's, and then the day was done. A pleasant day in spite of fog and cold, for the gratitude of matrons and sisters was very warm and bright, and we pass it all on to our readers, with our own hearty thanks for their co-operation with our scheme.

### National Pension Fund for Nurses.

### A NEW YEAR'S GIFT.

EXPERIENCE has shown that a large number of nurses have hesitated to join the Fund because they could not use it as a savings bank; or, in other words, because they were not allowed interest on their premiums when obliged to withdraw them. Private nurses have also had considerable difficulty in placing their savings in the Fund, because they feared they might be unable, sooner or later, to continue regular payments. The history of many cases prove that these objections were well founded, and after full consideration the Council have determined to grant the following additional privileges to policy-holders on and after January 1st, 1891:—

(1) Two and a-half per cent. interest will be allowed upon all returnable premiums withdrawn, less the cost of administration, to be ascertained in consultation with the actuary.

(2) In order to meet the special difficulties of private nurses in regard to regular fixed monthly or periodical payments, nurses will henceforth be permitted to pay into the Fund, irregularly, such amounts as they may be able to deposit with it. Directly these deposits amount to the sum of £10, they will be applied to the purchase of a paid-up policy in the Fund on the returnable scale, or to the payment of premiums on fixed policies, as each nurse may prefer.

These concessions are most important, as they now make the Fund, so far as nurses are concerned, in practice a savings bank, with all the advantages of the Post Office Savings Bank, plus the privilege of participating in donation and profit bonuses from time to time as they may be declared by the Council. The Donation Bonus Fund now amounts to £40,000, and the Junius S. Morgan Benevolent Fund to £10,000, and these large funds, combined with the new privileges just granted by the Council, make the National Pension Fund for Nurses not only a tower of strength and a good business investment for all savings, but a safe anchorage for nurses in the day of sickness or incapacity of any kind. New policies have been issued throughout the year at the rate of more than one each day, and henceforward the number of members who join will no doubt be indefinitely multiplied.

A general scheme of affiliation for hospitals is also in preparation, and there is good reason to hope that no well-managed hospital or nursing institution will long remain unaffiliated to the Fund on the half-premium principle, under which the institutions pay half the premium of every nurse in their employ who joins the Fund.

### Presentations.

THE Lady Superintendents of the Blackheath and Richmond Nursing Institutions have been presented by their nurses with a very handsome pair of fish servers as a Christmas present. They are much gratified, not only because of the beauty and value of the gift, but by the kind feeling which prompted it.

Christmas morning was made the occasion of a pleasant and well-deserved surprise for the Matron (Mrs. Phillips) of Queen Charlotte's Hospital. The pupil midwives and nurses presented her with a handsome pair of bronze horses, which were subscribed for by them, and accompanied by each one's signature. The Matron was greatly pleased and much affected. The Secretary, Mr. G. Owen Ryan, warmly thanked the nurses on the Matron's behalf in a few appropriate words.

On Christmas Day the sisters of the Western Infirmary, Glasgow, presented their matron, Miss Clyde, with a handsome case containing brushes—a slight memento of the esteem and affection in which she is held.

### Everybody's Opinion.

*[Correspondence on all subjects is invited, but we cannot in any way be responsible for the opinions expressed by our correspondents. No communications can be entertained if the name and address of the correspondent is not given, or unless one side of the paper only be written on.]*

### DIPHTHERIA AND THE USE OF BRANDY.

"VERITAS" writes: It appears that Dr. Carpenter's experience of nurses must have been very unfortunate when he states (according to your issue of the 29th ult.) that where brandy is used for diphtheretic patients it is also freely partaken of by the nurses, on the supposition that it is a protection against the insidious growth of the mysterious disease. When he said that cases were almost certain to end fatally where brandy was used, did he mean that the brandy killed the patients, or that the patient's life was sacrificed owing to the neglect of the nurse, subsequent to her having too freely imbibed the brandy herself? My own experience as a nurse is that brandy is an invaluable medecine in any fever, and I attribute the saving of scores of lives to its judicious use. I remember nursing a very bad case of diphtheria, where the parents of a boy were averse to the use of brandy, but the doctor overruled their prejudice; the patient had one teaspoonful of brandy every hour, he recovered, and there was no *sequelæ*. Some time ago I was alone in a country place with a typhoid case. On the eighth day, at 11 p.m., excessive hæmorrhage set in, and continued at intervals until 4 a.m.; the patient was in a state of collapse, and I had no means of communicating with anyone; it is my firm belief that without brandy a life would have been lost.

### SCOTCH INFIRMARIES.

LIEUT.-COLONEL E. MONTEFIORE writes: "The report given by you of my remarks at the general meeting of the Hospitals Association, held at the Middlesex Hospital on the 10th inst., when Miss Wilson read her interesting paper, is so very misleading that I should be glad if you would allow me a word of explanation. The picture I drew was of only one Scotch poor-house hospital, or poor-law infirmary (as we Southerners call it), which I had visited. I did not refer to the Scotch infirmaries, or hospitals as we should call them. It would seem from your note of what I said that I had referred to both classes of institutions, and not to one particular institution in one class.

### FEVER HOSPITALS.

MISS JESSIE ANDERSON writes from the Homerton Hospital:—It is to be hoped that all parties will make a thorough enquiry as to the alleged mismanagement of this institution. Perhaps then D. Halkin will not come out with such flying colours. I venture to say that I consider the courageous nurses are those who are still working faithfully at their posts. As I have been here three years I know both sides of the question.

"ROSE" writes: As correspondence is invited, I may be able to set "Thistle's" mind at rest on one or two points. I happen to know the circumstances of the case stated by her; for at that time I was one of the nurses in that particular hospital. I was not the only one who felt indignant at the treatment of a "sister nurse," whom anyone would be glad to see, for I may say "fresh faces" are a thing almost unknown there, except as patients, or a new nurse replacing an old one, which is a common occurrence. As regards "the old lady," I may say we did all in our power to make her happy and comfortable, and one nurse was particularly kind to her in reading and other attentions. The nurse's place is not always enviable, and her sleep is not always peaceful for more than one reason. I think the Committee must be asleep or easily hoodwinked not to see and hear from nurses themselves, that they at least have necessary things. I should have written to this page before, but that I dislike to be amongst the "grumblers," although my plea for the nurses is an honest one. However, as "Thistle" has kindly opened a channel, I see no reason why I should not help to widen it.

### THE TRAINED NURSE IN THE NURSERY.

"A FELLOW WORKER" writes: Whilst Sister Grace is on the subject of "The Trained Nurse in the Private Nursery" will she kindly give her opinion as to whether gentlewomen or those taken from the ordinary servant class make the better nurses? This is a question I am constantly being asked, and though I have had many years of experience can never answer to my own satisfaction. Allow me to state one or two ideas I hold on the matter and then if I am not correct, Sister may possibly set me straight. 1st. If gentlewomen are to be found in the nursery, mothers will be there less than ever, feeling that they can, with safety, throw off the responsibility of motherhood on to the shoulders of the better educated and more refined woman. 2nd. I do think a head nurse should be a mother and one who has taken a mother's share of work in her own home—she has then naturally studied the different temperaments of children and can better sympathise with and soothe the baby troubles of nursery life. I have met many single gentlewomen who have lovingly helped their own mother to bring up the younger children in their own family, and these in my opinion come next to mothers to fill the post of head nurse. It is unfortunate that a gentlewoman finds it go so against the grain to be patronised by so many she has to do with in a private nursery. This she never has to put up with in her work as a trained nurse. This, at least, is my experience.

### Appointments.

CARLISLE INFIRMARY.—Miss Augusta Margarite Orchard has been elected Superintendent of Nurses at the Fuse Hill Infirmary, Carlisle, out of 67 candidates. Miss Orchard won very high certificates for medical, surgical, and maternity nursing at the Chorlton Union Hospitals, Withington, where she trained, and where she has had charge of a pavilion for some time. Her testimonials are very good, and we wish her success, and congratulate her on her promotion.

VICTORIAN EYE AND EAR HOSPITAL.—Miss M. E. Stephenson has been appointed Lady Superintendent of this Melbourne hospital. Miss Stephenson trained at Crumpsall Infirmary, was charge nurse at the Haverstock Hill Fever Hospital, and did some private nursing in London and at the Isle of Wight. In 1889 Miss Stephenson emigrated to Australia, joined the St. Hilda Home, and finally was appointed sister of the male wards of the Launceston Hospital. Miss Stephenson holds excellent testimonials, and seems a general favourite; we congratulate her on her success.

### Notes and Queries.

### Queries.

(23) *Nursing Institutions.*—The question has been raised as to the desirability of establishing a school for nurses in connection with the East Suffolk and Ipswich Hospital. I should be glad to know the names of some of the hospitals which have similar institutions connected with their hospital, also on what principles they are worked, and if they are generally a source of profit or loss. Any information through your paper, or sent direct to me, I shall be very thankful for.—SECRETARY.

(24) *District Nurse.*—What remuneration should a district nurse receive?—*J. H. C.*

### Answers.

*District Nurse.*—£80 is not too much a year for a district nurse. At Sutton, £100 a year is reckoned as the cost of such a nurse, the extra money going in appliances, nourishment, and clothing for the sick.

*Nursing of Children.*—We beg to inform "A. D." that she will find a chapter on nursing sick children in "Lectures on Nursing," by Miss Luckes, published by Kegan Paul, price 2s. 6d. Miss Wood's book is published by Cassell and Co. "The Management of Children," published by Churchill, is a good book, but rather bulky and expensive.

*M. G.*—Sorry we have no room for the verses sent.

(20) *Percentage.*—The private nurses at the Middlesex and at St. Bartholomew's receive a percentage on their earnings. Doubtless particulars could be had on application.—*N. O.*

*J. F.*—Your letter has been forwarded to Miss Emily Jones, who offered the books.

### The Brimsby Ghost.

At last, there came a lull. I, Gertrude Trent, had become worn-out by a long spell of nursing. I had fought through three consecutive private cases; but, just before Christmas, I turned up at the institution. Possibly, I did not look particularly robust, for the holiday I requested was readily accorded.

"So we're to go down to Brimsby for Christmas, Gerty!" was my young brother's greeting when I looked him up at Dr. Birchem's. It was Frank's last term at school, and he was panting, with the eagerness of sixteen, to face the world.

"Yes, boy;" answered I, "the other Trents are anxious to have us; and its the only home-place where you and I could be together," for Frank and I are orphans.

"Old Peter Dixon said I might spend the holidays with him—but he didn't mention you, Gerty."

"Old Peter shan't have you," I spoke jealously; "the other Trents want us both. And hadn't you better say 'Mr. Dixon,' now, seeing he'll shortly be your chief?" For facing the world, to Frank, meant from a stool in the firm of Dixon and Co.

"Bother!" said the boy, "Look here, Gerty, we're going to have a high old time at Brimsby. We'll sink Dixon and Co., and as for Nurse Trent, here she goes!"

There was a sudden collapse of my headgear; I was suffocated and blinded; then, to my dismay, there was my bonnet and veil rolled up in the cloak I had removed on entering and Frank coolly footballing the bundle.

"Oh, Frank"!

"Well, what does it matter? You're not going down to Brimsby in these articles. You'll wear a decent hat and a smart jacket, if you please, Miss Trent. A man likes to have his women-folk resemble other people when he takes them out."

The "man" looked so comical that I had to laugh, though the shaping straight of that bonnet was not amusing.

The whole earth seemed ice bound as the train that took us to Brimsby thundered along. Thankful enough were we when it creaked into the small country station.

"There's Old Noll come for us, in the trap!" cried Frank irreverently.

The "other Trents" our cousins, are as well off as we are impecunious. Both our fathers are dead, but Aunt Sophy has plenty of money; she and the girls live at the Rectory-house, with the Rev. Oliver, one of the best of sons. From out their world, full of its comforts and ease, the other Trents look, with pitying eyes, upon my hard working career. They would gladly give me a corner in their home, but I am not cut out, somehow, by nature for a dependent life.

There was no mistake about the warmth of our welcome when the Rev. Oliver deposited us at the hall-door of as queer, overgrown, and comfortable a house as ever was built, and added to—principally added to.

"*This* is something like keeping Christmas," said Frank approvingly, as we stood in the large hall, a log crackling in its roomy fire-place, and the flickering flames picking out the red holly berries from the dim corners.

"Now that we have you, my dears," said Aunt Sophy, her arms round us both, "it will really be Christmas."

"A houseful of Trents!" laughed Milly, the eldest girl; "hope you won't be disappointed to hear we've nobody but ourselves!"

"And our Ghost," put in Oliver.

"Halloa! A ghost?" Frank's eyes jumped; "That's a lark! Has he come to stay?"

But we heard no more about that visitor until dinner was over, and we were seated in orthodox fashion encircling the fire.

"Our Ghost," began Milly lightly, while Nancy, the younger girl, a fantastic, dreamy maiden, shivered, and Oliver looked serious, "is a Presence that visits the study, nightly, sits in Noll's chair, and reads his book."

(*To be continued.*)

### Where to Go.

On January 13th at half-past eight p.m. will be performed, at St. George's Hall, "The Golden Apple," a mythological drama. The performance is in aid of the Kate Marsden Nurse Fund, and the front row of the balcony stalls, at two shillings each, have been reserved for nurses, whom, it is hoped, will appear in uniform. Application for tickets should be made at once to Mrs. Brewer, 21, George Street, Hanover Square, W.

At the College for Working Men, Great Ormond Street, there will be free popular lectures (women admitted) on Saturdays at half-past eight p.m. On January 17th, by Herbert Bayne, M.A., on "Beethoven"; on January 24th, by Percy Fitzgerald, M.A., "Recollections of Dickens"; on January 31st, by Professor J. Westlake, Q.C., M.A., on "The Theory and Laws of War."

At the Royal Institution, Albermarle Street, at three p.m., Professor Victor Horsley, F.R.S., B.S., F.R.C.S., M.R.I., Fullerian Professor of Physiology, R.I. Nine lectures on "The Structure and Functions of the Nervous System." Part I.: The Spinal Cord and Ganglia. On Tuesdays, January 20th, and 27th, February 3rd, 10th, 17th, and 24th, March 3rd, 10th, and 17th. One guinea the course.

### Amusements and Relaxation.

### SPECIAL NOTICE TO CORRESPONDENTS.

First quarterly word competition commences January 3rd, 1891; ends March 28th, 1891.

Three prizes of 15s., 10s., 5s., will be given for the largest number of words derived from the words set for dissection.

N.B.—Word dissections must be sent in WEEKLY not later than the first post on Thursday to the *Prize Editor*, 140, Strand, W.C., arranged alphabetically, with correct total affixed.

The word for dissection for this, the FIRST week of the quarter, being "HAPPY YEAR."

| Names. | Dec. 24th. | Totals. | Names. | Dec. 24th. | Totals. |
|---|---|---|---|---|---|
| Jenny Wren | 49 | 695 | Checkmate | — | 76 |
| Tinie | — | 55 | Silver King | — | 163 |
| Agamemnon | 54 | 718 | S. Anthony | — | 76 |
| Patience | 46 | 709 | Quackah | — | 75 |
| Ecila | 46 | 708 | Reynard | — | — |
| Lightowlers | 50 | 684 | Sally | — | 27 |
| Rouge | — | 89 | Success | — | 61 |
| Wyamaris | 55 | 709 | Caledonia | — | 52 |
| Qu'appelle | 50 | 611 | Nurse Emma | — | 305 |
| Nosam | — | — | Hazel | — | 20 |
| Nurse Hilda | — | 44 | Pallas | — | 48 |
| Lady Betty | 41 | 650 | Puss | — | 15 |
| Grenelle | — | 43 | Shakespeare | 20 | 575 |
| Daisy | — | 324 | Melita | — | — |
| H. A. S. | — | 157 | Nora | — | 10 |
| A. B. C. | — | 66 | Elsie | — | 35 |
| Liz | — | — | Espèrance | — | 27 |

### Notice to Correspondents.

N.B.—Each paper must be signed by the author with his or her real name and address. A *nom de plume* may be added if the writer does not desire to be referred to by us by his real name. In the case of all prize-winners, however, the real name and address will be published.

Competitors can enter for *all* quarterly competitions, but no competitor may take more than *one first prize* during the year.

## Figures and Tables

**Figure f1:**
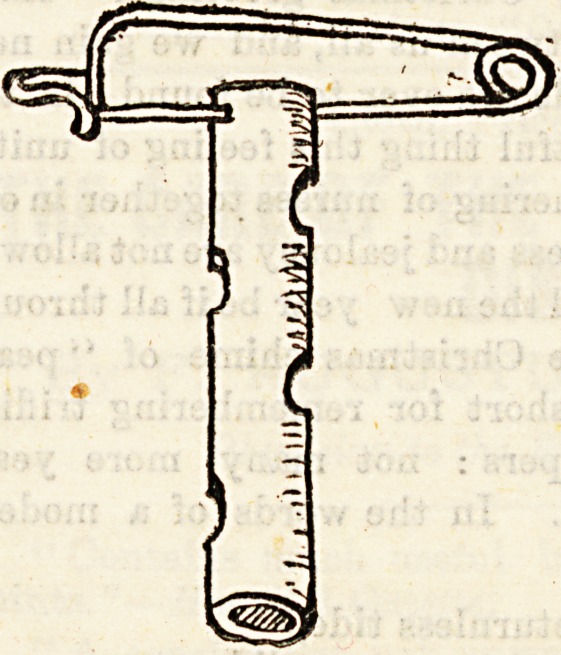


**Figure f2:**
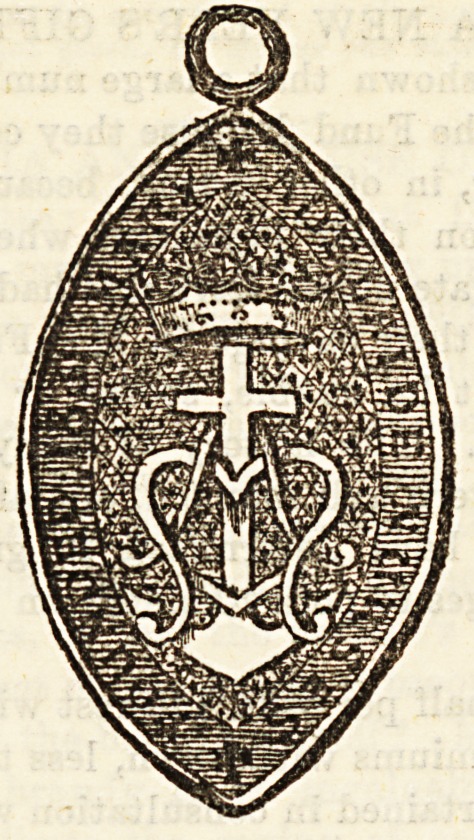


**Figure f3:**